# Recombinant SMN protein synergizes with spinal muscular atrophy therapy to counteract pathological motor neuron phenotypes

**DOI:** 10.1186/s40035-024-00455-4

**Published:** 2024-12-17

**Authors:** Liliana Brambilla, Chiara F. Valori, Giulia Guidotti, Francesca Martorana, Claudia Sulmona, Lisa Benedetta De Martini, Anselmo Canciani, Marco Fumagalli, Francesca Talpo, Gerardo Biella, Elisa Di Pasquale, Claudio Iacobucci, Federico Forneris, Haiyan Zhou, Daniela Rossi

**Affiliations:** 1 Laboratory for Research on Neurodegenerative Disorders, Istituti Clinici Scientifici Maugeri IRCCS, 27100 Pavia, Italy; 2 The Armenise-Harvard Laboratory of Structural Biology, Department of Biology and Biotechnology, University of Pavia, 27100 Pavia, Italy; 3 Department of Biology and Biotechnology, University of Pavia, 27100 Pavia, Italy; 4 Humanitas Cardio Center, IRCCS Humanitas Research Hospital, 20089 Rozzano, Milan, Italy; 5 Institute of Genetic and Biomedical Research (IRGB), UOS of Milan-National Research Council of Italy (CNR), 20138 Milan, Italy; 6 Department of Physical and Chemical Sciences, University of L’Aquila, 67100 L’Aquila, Italy; 7 Fondazione IRCCS Policlinico San Matteo, 27100 Pavia, Italy; 8 Dubowitz Neuromuscular Centre and National Institute for Health Research, Great Ormond Street Institute of Child Health, Biomedical Research Centre, University College London, London, UK

## Main text

Spinal muscular atrophy (SMA) linked to chromosome 5q is an autosomal recessive neuromuscular disease caused by mutations/deletions in the Survival Motor Neuron 1 (*SMN1*) gene, with consequent reductions of the level of the SMN protein. This results in spinal motor neuron degeneration and variable clinical presentations. The prognosis depends on the phenotypic severity, ranging from high mortality for the infantile form (SMA type 1) to no decrease of lifespan for the chronic and later-onset forms [1]. Humans possess a paralogous *SMN2* gene, which exhibits a single-nucleotide transition compared to *SMN1*, resulting in aberrant splicing of 80%–90% of *SMN2*-derived pre-mRNAs. It follows that *SMN2* produces minor amounts of full-length transcripts and primarily makes transcripts lacking exon 7 (*SMNΔ7*), thereby generating a SMN protein with reduced stability and only partial function. Thus, *SMN2* cannot fully compensate for the deficiency of *SMN1*, although its copy number inversely correlates with the severity of SMA.

In recent years, therapies aiming at raising SMN expression have become available, including the *SMN2* splicing modifiers nusinersen and risdiplam, and the onasemnogene abeparvovec viral vector for gene replacement therapy [1]. Although these revolutionary therapies have significantly improved motor and respiratory functions in SMA individuals, patients respond heterogeneously to these treatments with early intervention emerging as a key determinant for symptom improvement in clinical practice [2]. Among others, an important limitation of splicing modifiers is the amount of available *SMN2* pre-mRNAs, particularly in individuals affected by the most common and severe SMA form (type 1), who have typically only two *SMN2* copies, but likely require the highest and most rapid induction of SMN. This suggests that the timing and level of SMN expression are critical elements to attain treatment success. Yet, the intrinsic properties of existing therapies are such that they require time to achieve full transduction and cannot rapidly induce high SMN protein expression. Altogether, these observations suggest that existing treatments are still not a cure, highlighting the need for the development of additional approaches to improve the efficacy of these available therapies. Thus, we hypothesized that an easily deliverable, “ready-to-use” SMN protein could fulfill these requirements.

**Fig. 1:**
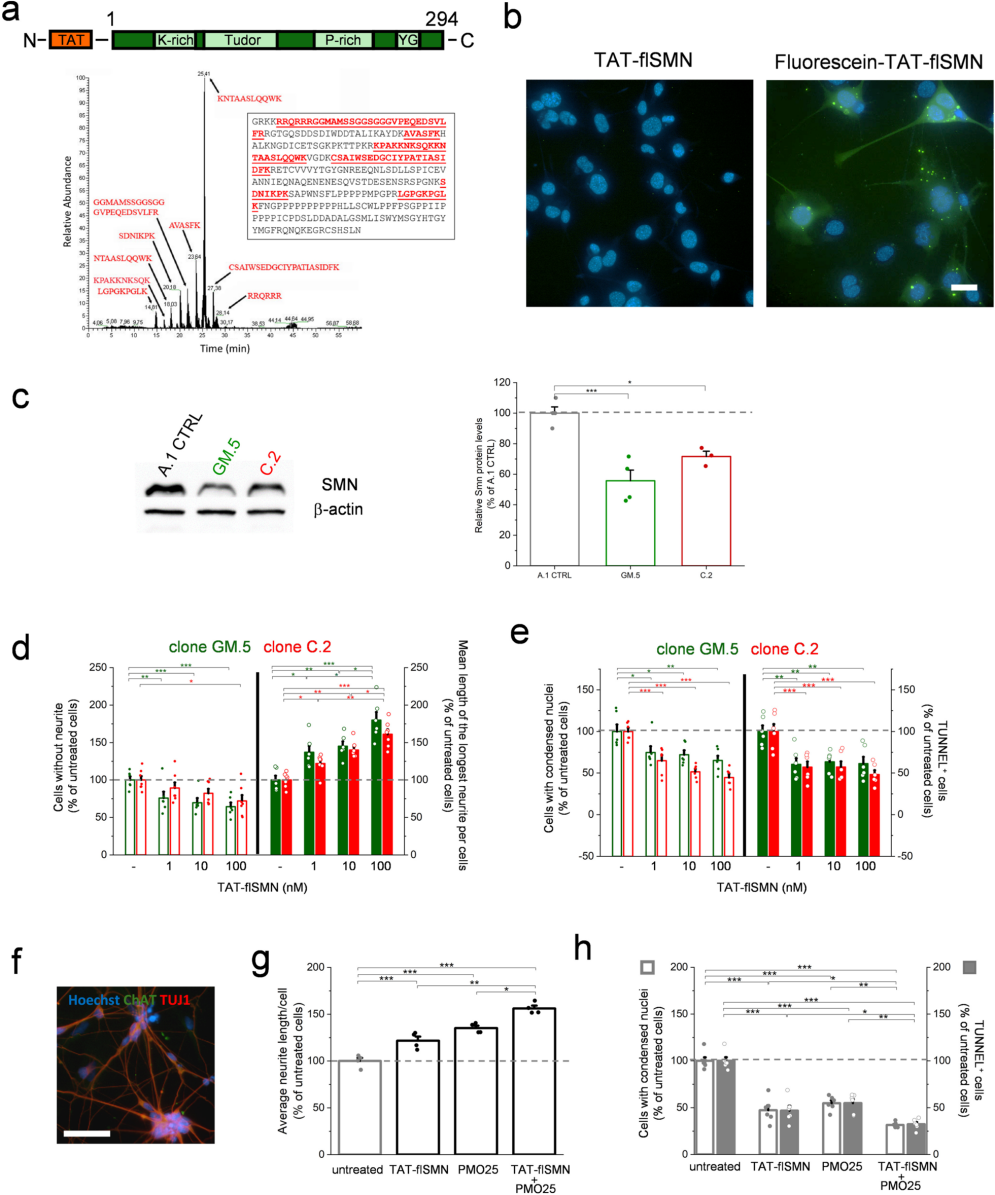
**a** Schematic presentation of recombinant TAT-flSMN protein topology and mass-spectrometry peak profile obtained from tryptic digestion of the sample. Inset shows the peptide sequence coverage (underlined) on the entire protein sequence. **b** Images show NSC-34 cells treated with 100 nM unlabelled (left) or Fluorescein-labelled TAT-flSMN (right, green). Nuclei were stained with Hoechst 33342 (blue). Scale bar, 50 μm. **c** Representative immunoblots (left) of SMN and β-actin proteins in the A.1 CTRL NSC-34 cells and the GM.5 and C.2 SMN-knockdown NSC-34 cell lines. Bands were quantified and SMN/β-actin ratios were calculated for normalization (right) (mean ± SEM; *n* = 3–4; *P < 0.05 and ***P < 0.001, Bonferroni-corrected one-way ANOVA). **d** Percentage of cells without neurites (left) and the mean length of the longest neurite per cell (right) in GM.5 (green) and C.2 (red) SMN-knockdown cells in the absence or presence of increasing concentrations of TAT-flSMN (mean ± SEM; *n* = 6–7; *P < 0.05, **P < 0.01, ***P < 0.0001, Bonferroni-corrected one–way ANOVA). **e** Percentage of cells with condensed nuclei (left) or showing active caspase 3 (right) in GM.5 (green) and C.2 (red) SMN-knockdown cells treated as in (**d**) (mean ± SEM; *n* = 6; *P < 0.05, **P < 0.001, ***P < 0.0001, Bonferroni-corrected one–way ANOVA). **f** Representative image of iPSC-derived MNs from a SMA patient stained with Hoechst 33342 and immunolabeled for beta III-tubulin (TUJ1) and choline acetyltransferase (ChAT). Scale bar, 50 µm. **g** Analyses of the average neurite length per cell in SMA iPSC-derived MNs treated with 10 nM TAT-flSMN, 0.5 μM PMO25, or 10 nM TAT-flSMN + 0.5 μM PMO25 (mean ± SEM; *n* = 4; *P < 0.05, **P < 0.01, ***P < 0.0001; Bonferroni-corrected one-way ANOVA). **h** Analysis of SMA iPSC-derived MNs with condensed nuclei or TUNEL^+^ staining upon treatment as in (**g)** (mean ± SEM; *n* = 6; *P < 0.05, **P < 0.01, ***P < 0.0001, Bonferroni-corrected one–way ANOVA)

In this study, we developed a recombinant full-length human SMN protein conjugated with the protein transduction domain of the HIV-1 trans-activator of transcription (TAT) protein (TAT-flSMN; Fig. 1a). The selection of the TAT domain was based on our previous observations that this peptide can promote the delivery of therapeutics to the central nervous system [3]. The precise identity of recombinant TAT-flSMN was confirmed by mass-spectrometry analyses (Fig. 1a). Furthermore, we thoroughly characterized the biophysical properties of this protein. A combination of differential scanning fluorimetry and size-exclusion chromatography coupled to small-angle X-ray scattering (SEC-SAXS) demonstrated that the TAT domain does not perturb proper SMN folding (Fig. S1). Furthermore, SAXS-based molecular weight analysis showed also the presence of large-molecular-weight species in solution (Fig. S1; Table S1), revealing that, similar to the native protein, recombinant TAT-flSMN undergoes self-oligomerization, a key property to ensure the biological function of SMN [4]. Besides, the TAT domain enabled protein internalization into murine NSC-34 motor neuron-like cells, as revealed by mass spectrometry analyses of cell lysates (Figs. S2-S4) and intracellular detection of fluorescein-labelled TAT-flSMN (Fig. 1b).

To investigate the biological activity of the fusion protein in vitro, we generated a cell model of SMA by short-hairpin RNA (shRNA)-mediated downregulation of the murine *Smn* gene expression in the NSC-34 cell line. Among cells transfected with the control shRNA, the A.1 clonal cell line was chosen as representative of control conditions (CTRL). In the group transfected with the *Smn*-specific shRNA, two SMN knock-down clonal cell lines, named GM.5 and C.2, exhibited a significant reduction of SMN protein level (30% to 40% reductions) compared to the A.1 CTRL, as assessed by Western blot analyses (Fig. 1c). Previous observations indicate that axon outgrowth and synapse formation/maintenance are impaired in cellular and animal models of SMA [5, 6]. At later stages of the disease, even motor cells that have developed normally may go through a degenerative process driven by apoptotic mechanisms [7]. To determine whether the GM.5 and C.2 SMN-knockdown cell lines reproduce these pathological events, we performed morphometric and cell death analyses. In the SMN-knockdown cells, we observed decreased neurite outgrowth/elongation (Fig. S5a) as well as enhanced susceptibility to apoptotic cell death, assessed as an increase of condensed nuclei and caspase-3 activation (Fig. S5b). These results suggest a defect in neurite outgrowth commitment and an impact on cell death that strictly correlate with the level of SMN expression, confirming the suitability of this cellular model of SMA for testing the effects of our therapeutic molecule. Next, we investigated whether TAT-flSMN administration could rescue these phenotypes. We found that the protein significantly counteracted the defects in a dose-dependent manner (Fig. 1d, e), thereby posing the basis for testing the efficacy of this agent in precision SMA disease models, both individually and in combination with other therapeutics already in clinical use.

Nusinersen, an antisense oligonucleotide (ASO) targeting the intronic splicing silencer N1 (ISS-N1), was the first drug to demonstrate that manipulating *SMN2* pre-mRNA splicing can result in enhanced production of full-length SMN protein and clinical efficacy [8]. Thus, in subsequent experiments, we tested an ISS-N1-targeting morpholino ASO, named PMO25 [9], for its capacity to abolish *SMN2* exon 7 skipping and to increase the levels of full-length SMN protein derived from *SMN2.*

We confirmed that PMO25 can promote exon 7 inclusion into *SMN2* transcripts and increase SMN expression in a dose-dependent manner in different experimental settings. These include (1) a cell-based luciferase assay performed on NSC-34 cells transfected with mini-genes that express the SMN-luciferase fusion protein under the control of the human *SMN1* or the *SMN2* promoter, respectively, and include the exon 7 splicing cassette (Fig. S6a), and (2) RT-qPCR analysis of full-length *SMN2* and *SMNΔ7* mRNA expression levels in SMA patient fibroblasts (Fig. S6b). Having observed remarkable beneficial effects of TAT-flSMN and PMO25 in increasing the expression levels of SMN as well as in improving morphometric features and recovering vitality in NSC-34 cells, we further investigated the impact of the individual and combinatorial administration of these two molecules on a patient-related neuronal model. We obtained inducible pluripotent stem cells (iPSCs) from a healthy SMA carrier (CTRL) and from a related SMA type 2 patient. We demonstrated that we could generate cells exhibiting molecular markers (Fig. 1f; Fig. S7a) and electrophysiological properties of mature motor neurons (MNs) (Fig. S7b-k). Besides, we showed that CTRL and SMA iPSCs differentiated into MNs with the same efficiency (Fig. S8a). Yet, SMA MNs exhibited reduced neurite elongation (Fig. S8b) and increased susceptibility to apoptotic cell death (Fig. S8c). Next, we determined that the individual administration of increasing concentrations of TAT-flSMN (1–100 nM) or PMO25 (0.5–2.5 µM) significantly ameliorated these phenotypes (Fig. S9). Notably, the effects of PMO25 on SMA iPSC-derived MNs rapidly reached saturation in the dose–response evaluation (Fig. S9b, d). One possible explanation is the limited amount of *SMN2* pre-mRNA in the SMA patient iPSC-derived MNs due to the low number of copies of *SMN2* gene. In addition, it has been recently demonstrated that the ASO therapy can induce an epigenetic block on *SMN2*, thereby limiting its transcription [10]. Remarkably, the concomitant administration of TAT-flSMN with PMO25 had synergistic beneficial effects on iPSC-derived MNs (Fig. 1g, h), suggesting that a timely SMN boosting can greatly ameliorate pathological motor neuronal phenotypes in SMA. Altogether, these results provide compelling proof-of-principle evidence of the therapeutic potential of our recombinant protein as an add-on to the existing therapies. We envision that the clinical management of SMA will improve with more refined strategies to raise SMN expression, such as TAT-flSMN, but also by associating them with nutritional and rehabilitation programs in an all-encompassing patient care plan.

## Supplementary Information

**Additional file 1**. **Figure S1**. Evaluation of recombinant TAT-flSMN folding and oligomerization states. **Figure S2**. Deconvoluted MS/MS spectrum of the 2+ charged ion at m/z 1156.5177 (precursor mass deviation: -0.7 ppm; retention time: 110.36 min). **Figure S3**. Deconvoluted MS/MS spectrum of the 3+ charged ion at m/z 887.4210 (precursor mass deviation: 2.4 ppm; retention time 198.2 min). **Figure S4**. Deconvoluted MS/MS spectrum of the 3+ charged ion at m/z 585.6333 (precursor mass deviation: -2.7 ppm; retention time: 36.359 min). **Figure S5**. Effect of SMN depletion on neurite outgrowth and cell death in differentiated NSC-34 motor neuronal cells. **Figure S6**. Impact of PMO25 on NSC-34 cells expressing SMN-reporter mini-genes and human SMA fibroblasts. **Figure S7**. iPSC differentiation into mature, electrophysiologically active motor neurons. **Figure S8**. Impact of SMN deficiency on SMA iPSC properties. **Figure S9**. TAT-flSMN and PMO25 induce neurite elongation and prevent apoptotic cell death on SMA iPSC-derived MNs. **Table S1**. Summary of SAXS data analysis. **Supplementary Materials and Methods**.

## Data Availability

All data generated or analyzed during this study are included within the article and the additional files.

## Abbreviations

SMA: Spinal muscular atrophy
SMN1: Human survival motor neuron 1
SMN2: Human survival motor neuron 2
SMNΔ7: Human SMN2 lacking exon 7
TAT: Trans-activator of transcription
SEC-SAXS: Size-exclusion chromatography coupled to small-angle X-ray scattering
shRNA: Short hairpin RNA
ASO: Antisense oligonucleotide
ISS-N1: Intronic splicing silencer N1
RT-qPCR: Reverse transcription quantitative polymerase chain reaction
iPSC: Induced pluripotent stem cell
MN: Motor neuron
