# Supplementary material for: Recombinant SMN protein synergizes with spinal muscular atrophy therapy to counteract pathological motor neuron phenotypes
Source: Transl Neurodegener. 2024 Dec 17;13:63. doi: 10.1186/s40035-024-00455-4 (PMC11650830; doi:10.1186/s40035-024-00455-4)
Supplement: Supplementary file 1 — Additional file 1. Figure S1. Evaluation of recombinant TAT-flSMN folding and oligomerization states. Figure S2. Deconvoluted MS/MS spectrum of the 2+ charged ion at m/z 1156.5177 (precursor mass deviation: -0.7 ppm; retention time: 110.36 min). Figure S3. Deconvoluted MS/MS spectrum of the 3+ charged ion at m/z 887.4210 (precursor mass deviation: 2.4 ppm; retention time 198.2 min). Figure S4. Deconvoluted MS/MS spectrum of the 3+ charged ion at m/z 585.6333 (precursor mass deviation: -2.7 ppm; retention time: 36.359 min). Figure S5. Effect of SMN depletion on neurite outgrowth and cell death in differentiated NSC-34 motor neuronal cells. Figure S6. Impact of PMO25 on NSC-34 cells expressing SMN-reporter mini-genes and human SMA fibroblasts. Figure S7. iPSC differentiation into mature, electrophysiologically active motor neurons. Figure S8. Impact of SMN deficiency on SMA iPSC properties. Figure S9. TAT-flSMN and PMO25 induce neurite elongation and prevent apoptotic cell death on SMA iPSC-derived MNs. Table S1. Summary of SAXS data analysis. Supplementary Materials and Methods. [file 40035_2024_455_MOESM1_ESM.pdf]

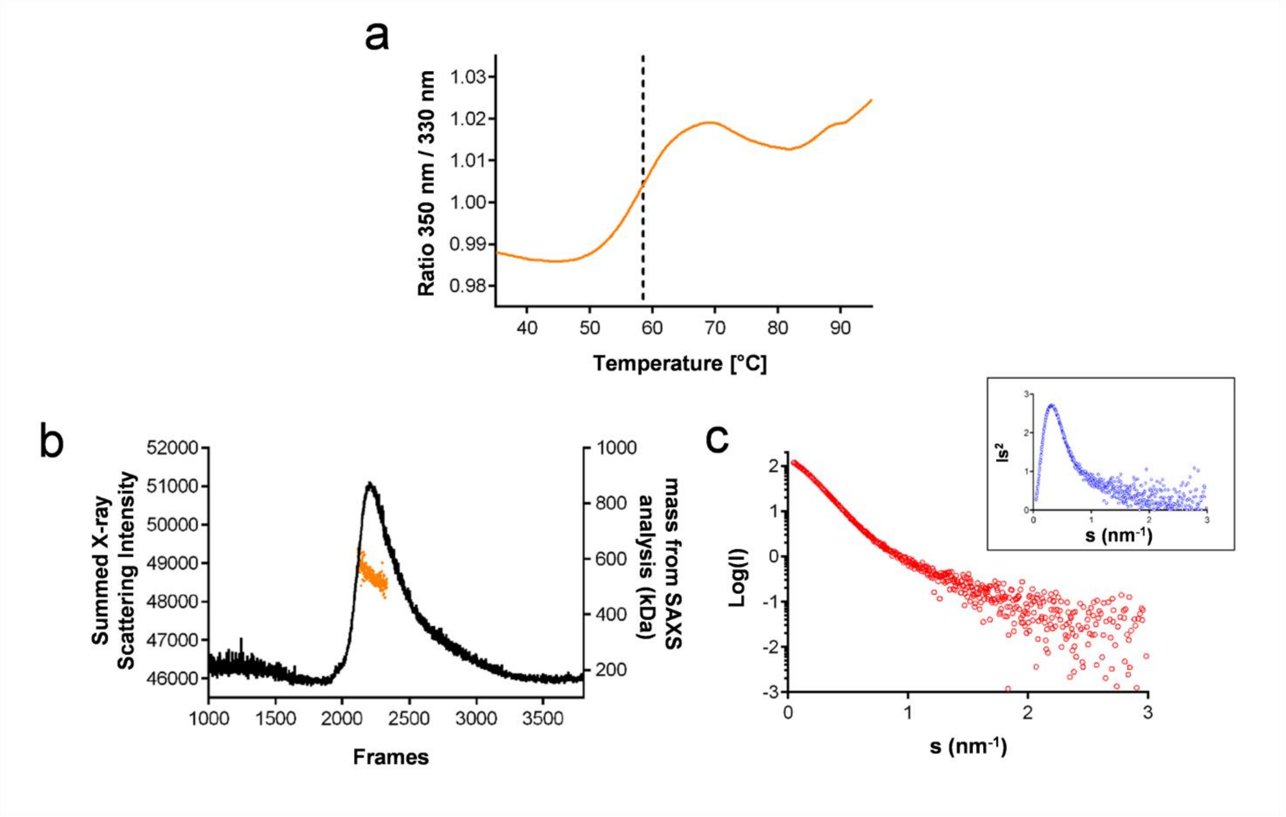


**Fig. S1. Evaluation of recombinant TAT-flSMN folding and oligomerization states. a.** DSF analysis of recombinant TAT-flSMN. The presence of an unfolding transition profile ($T_u$ = 58.5 °C) confirms the folded state of the sample tested. **b.** SEC-SAXS chromatogram obtained from the injection of 50µl of recombinant TAT-flSMN onto a Superdex 75 PC 3.2 column. Showed is the summed X-ray scattering intensity (black line) of SAXS frames recorded using an exposure time of 1 second per frame at ESRF BM29. Orange dots indicate the molar mass of the sample eluting in the peak region as computed using CHROMIXS. **c.** SAXS curve (red dots) derived from SEC-SAXS analysis of the peak region. Inset shows the corresponding Kratky plot (blue dots), highlighting the bell-shaped peak at low s values, indicative of a well-folded sample. SAXS-based molecular weight analysis of the single SEC peak also showed the presence of large molecular weight (480 kDa) species in solution. Details of SEC-SAXS data analysis are summarized in Table S1.

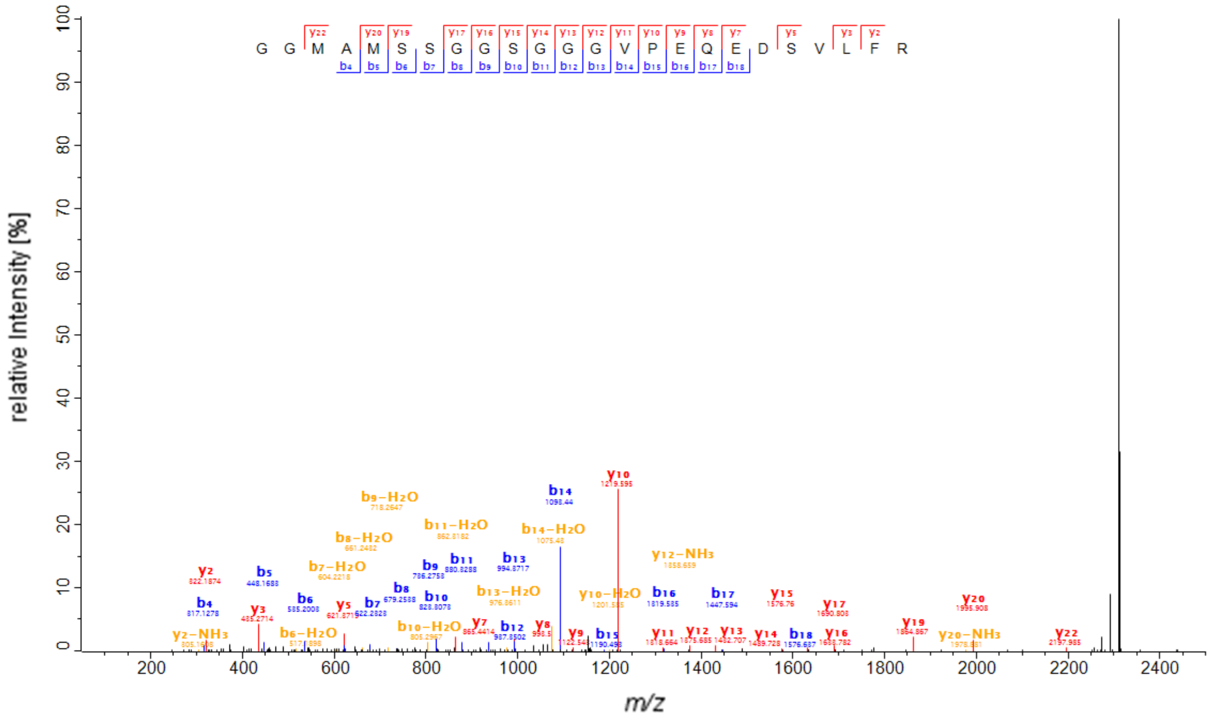


**Fig. S2. Deconvoluted MS/MS spectrum of the 2+ charged ion at m/z 1156.5177 (precursor mass deviation: -0.7 ppm; retention time: 110.36 min).** NSC-34 cells were treated with 100 nM TAT-flSMN for 48 hours, washed and lysated. Cell lysates were proteolyzed and analysed by mass spectrometry. The spectrum in the image has been assigned to the unique TAT-flSMN peptide GGMAMSSGGSGGGVPEQEDSVLFR.

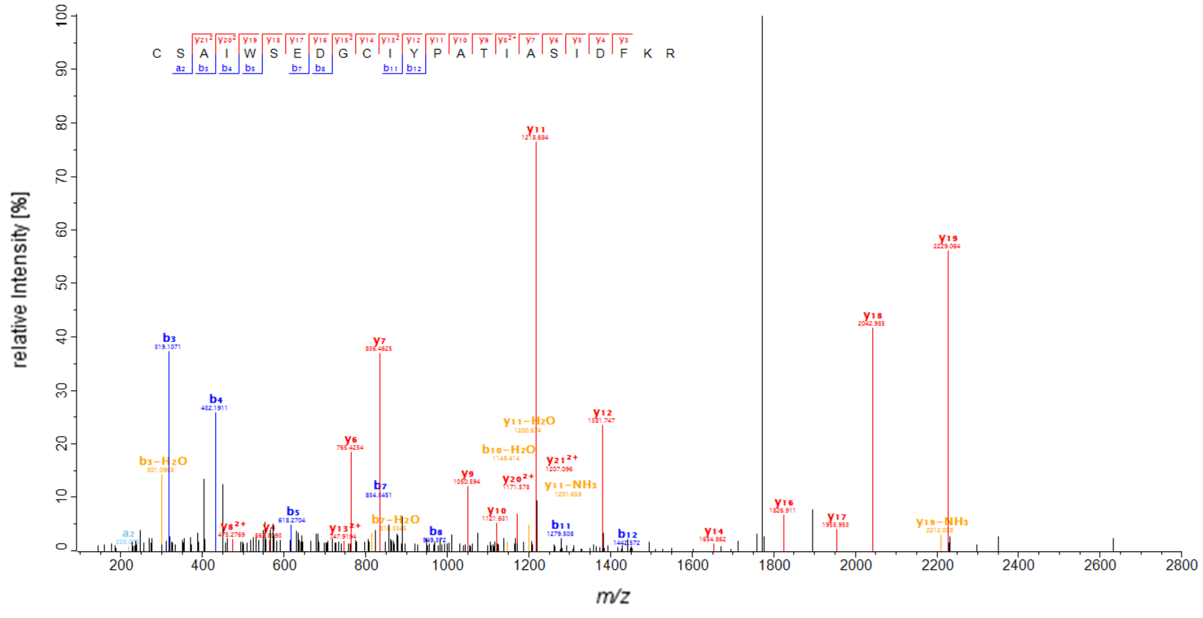


**Fig. S3. Deconvoluted MS/MS spectrum of the 3+ charged ion at *m/z* 887.4210 (precursor mass deviation: 2.4 ppm; retention time 198.2 min)**. NSC-34 cells were treated with 100 nM TAT-flSMN for 48 hours, washed and lysated. Cell lysates were proteolyzed and analysed by mass spectrometry. The spectrum in the image has been assigned to the unique TAT-flSMN peptide CSAIWSEDGCIYPATIASIDFKR.

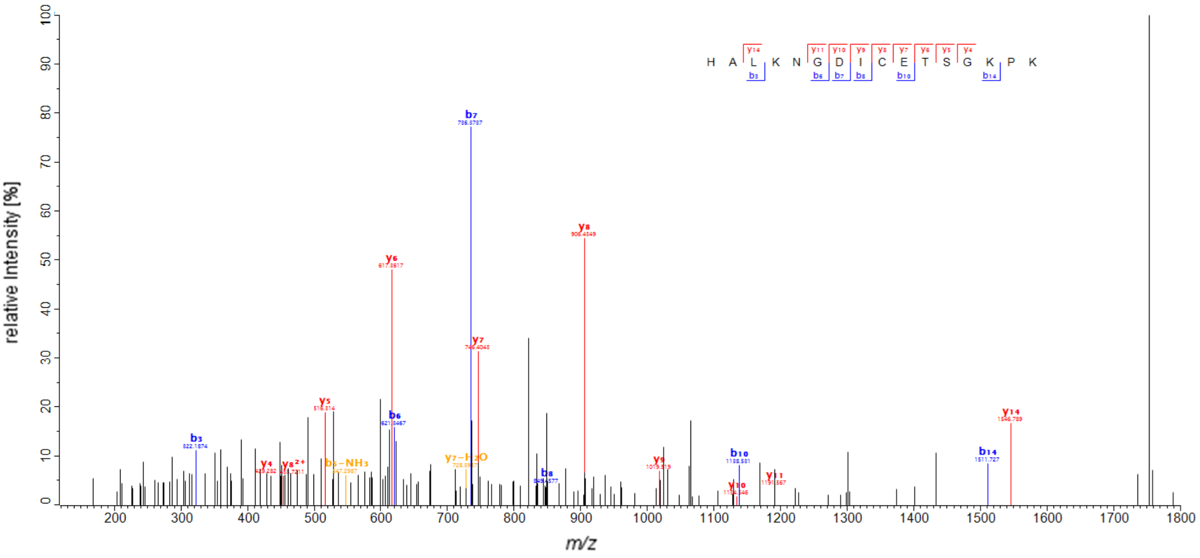


**Fig. S4. Deconvoluted MS/MS spectrum of the 3+ charged ion at m/z 585.6333 (precursor mass deviation: -2.7 ppm; retention time: 36.359 min).** NSC-34 cells were treated with 100 nM TAT-flSMN for 48 hours, washed and lysated. Cell lysates were proteolyzed and analysed by mass spectrometry. The spectrum in the image has been assigned to the unique TAT-flSMN peptide HALKNGDICETSGKPK.

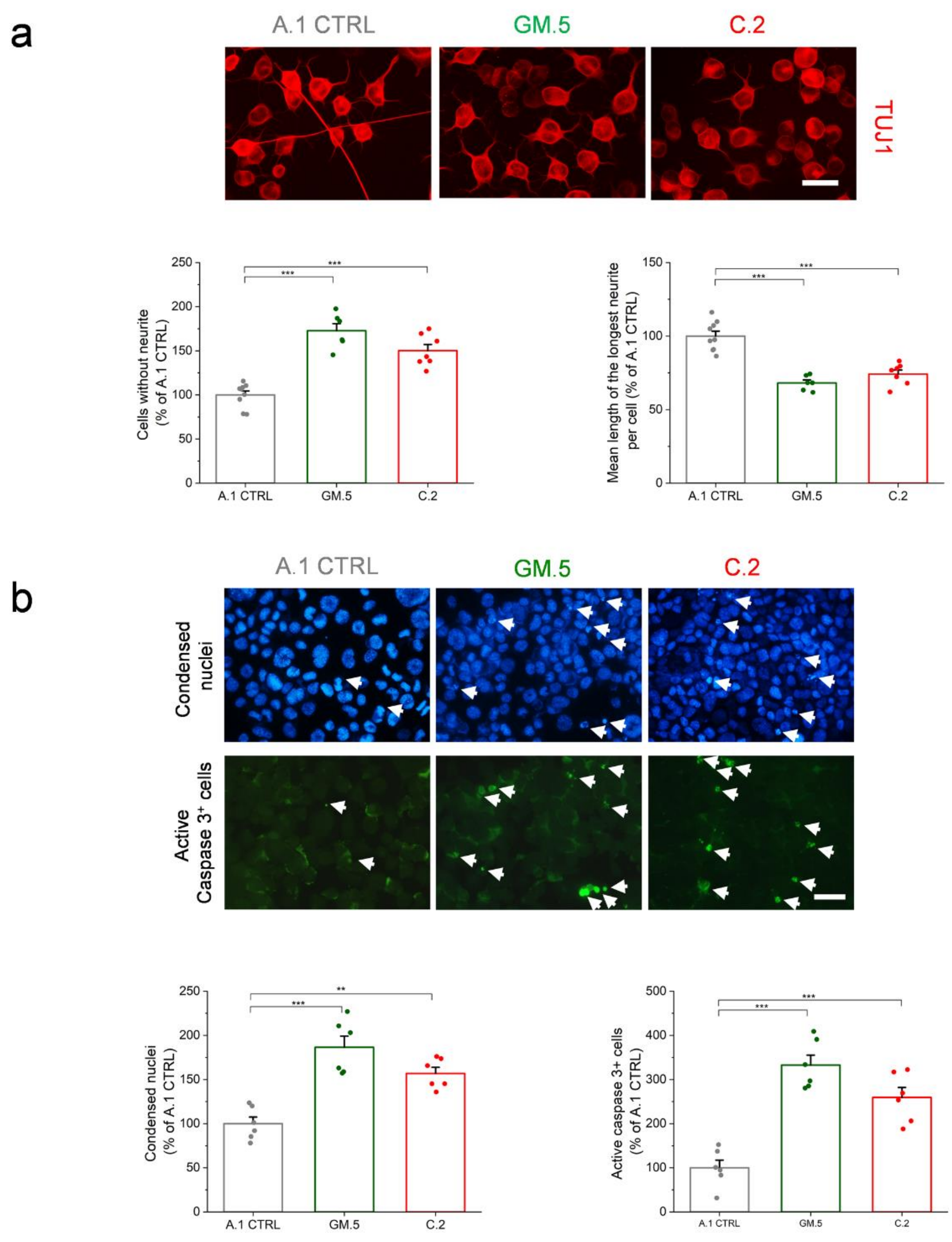


**Fig. S5. Effect of SMN depletion on neurite outgrowth and cell death in differentiated NSC-34 motor neuronal cells. a.** Representative images (*top*) of A.1 CTRL, GM.5 and C.2 cells immunolabelled for beta III-tubulin (TUJ1), 48 hours after the switch to differentiating conditions (DMed). Scale bar, 50µm. Morphometric analysis (*bottom*) revealed that SMN knock-down in GM.5 and C.2 cells significantly increased the number of cells without neurites and reduced the mean length of the longest neurite per cell. Data (mean ± SEM) are expressed as % in the A.1 CTRL cell line (% of cells without neurites: 15.48 ± 1.12%; mean length of the longest neurite per cell: 95.89 ± 4.18 µm; *n* = 6-9). ***p<0.0001, Bonferroni-corrected one–way ANOVA. **b.** Representative images (*top*) of A.1 CTRL, GM.5 and C.2 cells stained with Hoechst 33342 (blue, condensed nuclei) or NucView488 (green, active caspase 3). Arrows indicate condensed nuclei and cells with active caspase 3. Scale bar, 50µm. Histograms (*bottom*) indicate the quantification of the % of GM.5 and C.2 cells with condensed nuclei or active caspase 3. Data (mean ± SEM) are expressed as % of cells with condensed nuclei or active caspase 3 in the A.1 CTRL cell line (% of cells with condensed nuclei: 1.57 ± 0.19%; % of cells with active caspase 3: 0.71 ± 0.16%; *n* = 6). **p<0.01, ***p<0.0001, Bonferroni corrected one–way ANOVA.

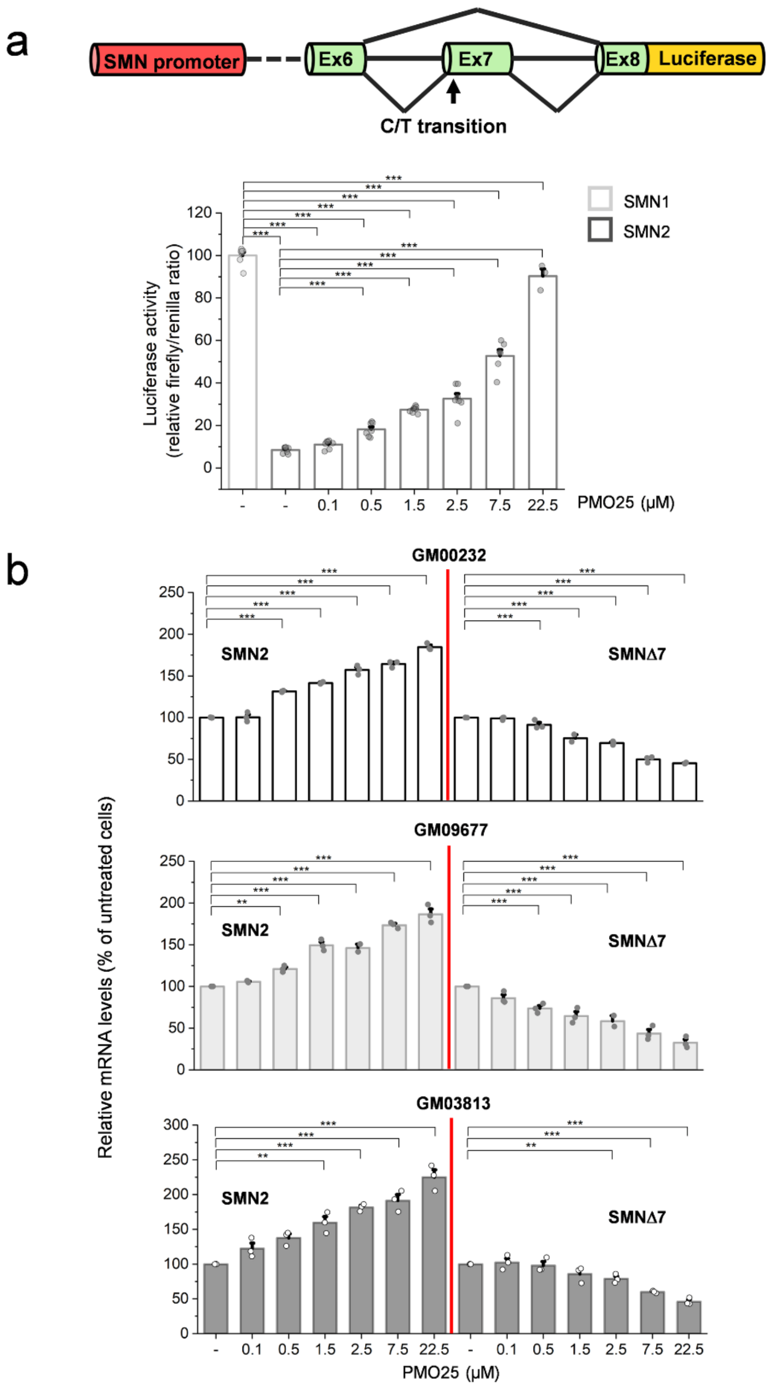


**Fig. S6. Impact of PMO25 on NSC-34 cells expressing *SMN*-reporter mini-genes and human SMA fibroblasts.** **a.** Top diagram illustrates the *SMN*-reporter mini-genes. The bottom histogram represents luciferase activity in NSC-34 cells transfected with the *SMN1*- and *SMN2*-reporter mini-genes and treated with increasing concentrations of PMO25. Data (mean ± SEM) are expressed as % of luciferase activity in untreated *SMN1*-reporter cells ($n$ = 3-7). ***p<0.0001, Bonferroni-corrected one-way ANOVA. **b.** Fibroblasts from SMA type 1 (GM00232, GM09677) and SMA type 2 (GM03813) patients were incubated with increasing concentrations of PMO25. Total RNA was extracted, reverse transcribed and analyzed by RT-qPCR. Data (mean ± SEM) are normalized relative to *HPRT* and expressed as percentage of *SMN2* or *SMNΔ7* mRNA levels in untreated patient-matched fibroblasts ($n$ = 3). **p<0.01, ***p<0.0001, Bonferroni-corrected one-way ANOVA.

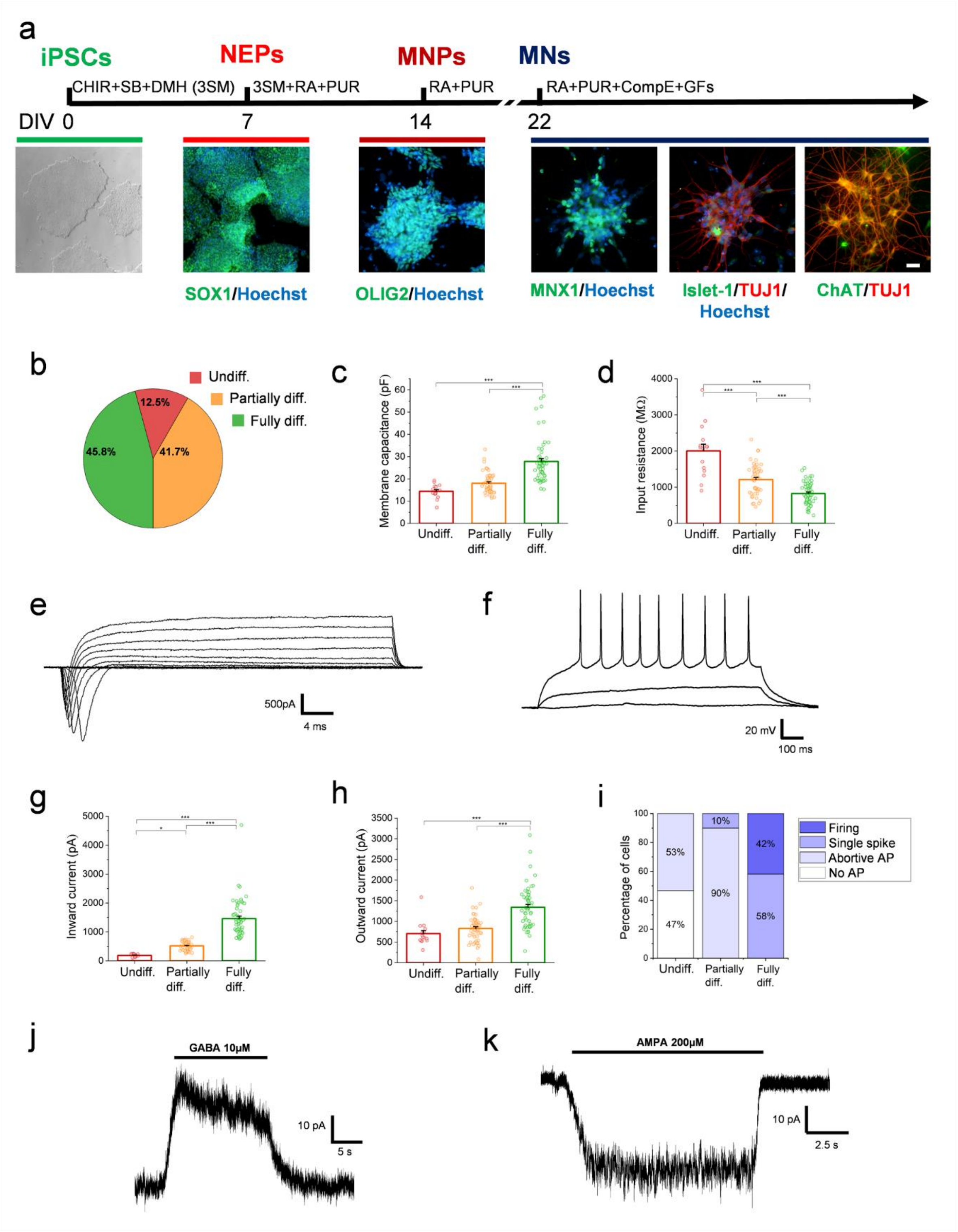


**Fig. S7. iPSC differentiation into mature, electrophysiologically active motor neurons. a.** Schematic diagram of the time-course and small molecules for differentiation of CTRL iPSCs (left brightfield image) into neuroepithelial precursors (NEPs), motor neuron progenitors (MNPs), and mature motor neurons (MNs). Under the time-line, representative images show each differentiation step as confirmed by immunocytochemical evaluation of stage-specific markers: SOX1 for NEPs; OLIG2 for MNPs; MNX1, Islet-1, TUJ1 and ChAT for MNs. Nuclei were stained for Hoechst 33342. Scale bar, 50μm. **b.** Percentage of cells subdivided accordingly to the maximal amplitude of their inward current peak: undifferentiated, with a negligible sodium peak (<250pA; n = 15/120; Undiff.); partially differentiated cells, with a sodium peak ranging from 250 to 750 pA (n = 50/120; Partially diff.); and fully differentiated cells, with a sodium peak greater than 750 pA (n = 55/120; Fully diff.). **c-d.** Histograms show the

differences observed in membrane capacitance (correlates with cell size) **(c)** and input resistance (correlates with the expression of leakage channels) **(d)** among undifferentiated, partially differentiated, and fully differentiated cells. (mean ± SEM; Undiff.: n = 15; Partially diff.: n =50; Fully diff.: n =55). **e.** Representative inward and outward currents recorded during a voltage-clamp experiment from a differentiated cell at test potentials ranging between –70 and +40 mV from a holding potential of –90 mV. Notice the presence of sizable inward (negative) and outward (positive) currents. **f.** Representative response of a differentiated cell to the injection of sub-threshold and supra-threshold depolarizing current steps. Notice the repetitive firing response to the supra-threshold step. **g-i.** Histograms show the differences observed in maximal inward **(g)** and outward currents recorded at +40 mV **(h)** between undifferentiated, partially differentiated, and fully differentiated cells. (mean ± SEM; Undiff.: n = 15; Partially diff.: n =50; Fully diff.: n =55). **i.** Percentage of cells subdivided accordingly to their ability to generate action potentials in each group (undiff. *vs.* partially diff. and *vs.* fully diff.). **j** Representative positive current deflection induced by the application of GABA 10 µM (black line) during a voltage-clamp experiment at a holding potential of 0mV in a differentiated cell. **k.** Representative negative current deflection induced by the application of AMPA 200 µM (black line) during a voltage-clamp experiment at a holding potential of -70mV in a differentiated cell. We found a similar response in 53% of the fully differentiated cells (10 out of 19) *p<0.05; **p<0.005; ***p < 0.001, Bonferroni-corrected one–way ANOVA.

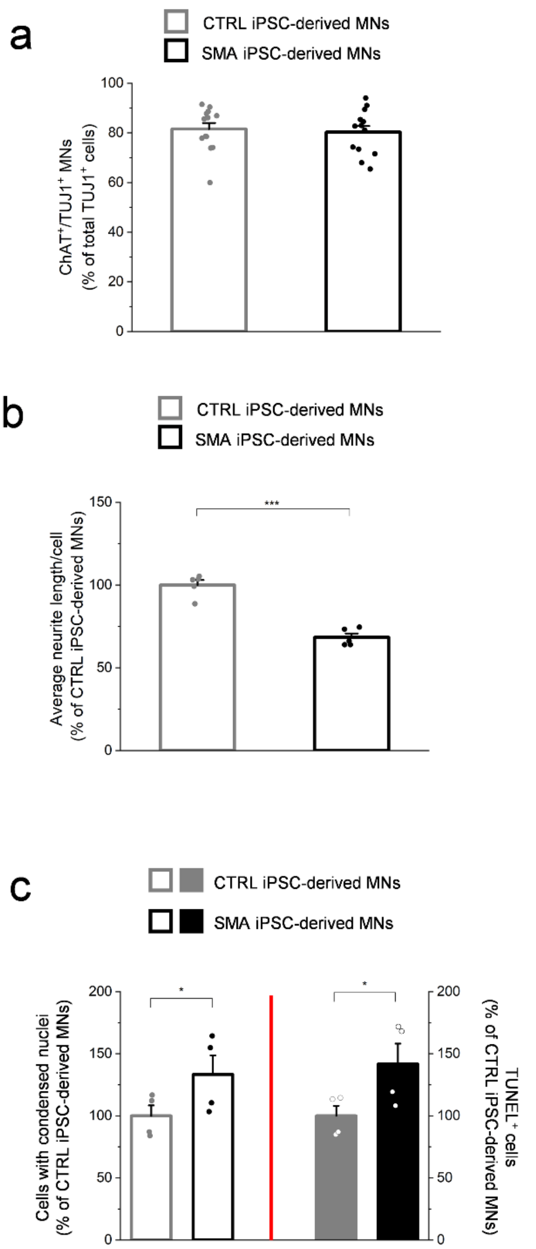


**Fig. S8. Impact of SMN deficiency on SMA iPSC properties. a.** Quantification of ChAT$^+$/TUJ1$^+$ immunopositive MNs obtained by differentiation of CTRL and SMA iPSCs at DIV29. Data (mean ± SEM) are expressed as % of ChAT$^+$/TUJI$^+$ MNs on the total TUJI$^+$ cells in 10 random fields per each line (*n* = 13). p = 0.3454, two-tailed unpaired *t*-test. **b.** At DIV29 of differentiation, CTRL and SMA iPSC-derived MNs were immunolabelled for TUJ1 and ChAT and subjected to morphometric analysis. Histogram indicates a significant reduction in the average neurite length per cell in SMA iPSC-derived MNs when compared to CTRL iPSC-derived MNs. Data (mean ± SEM) are expressed as % in CTRL iPSC-derived MNs (174.21 ± 5.67 µm; *n* = 5) ***p<0.0001, two-tailed unpaired *t*-test. **c.** At DIV29 of differentiation, CTRL and SMA iPSC-derived MNs were labeled with Hoechst 33342 to visualize condensed nuclei, with TUNEL to detect cells with fragmented DNA, and for TUJ1 to identify neurons. Quantification of cells with condensed nuclei and TUNEL$^+$ revealed a significant increase in cell death in SMA iPSC-derived MNs when compared to CTRL iPSC-derived MNs. Data (mean ± SEM) are expressed as % of cells with condensed nuclei or TUNEL$^+$ cells in CTRL iPSC-derived MNs (% of cells with condensed nuclei: 4.93 ± 0.46%; % of TUNEL$^+$ cells: 4.31 ± 0.40%; *n* = 4). *p<0.05, ***p<0.0001, two-tailed unpaired *t*-test.

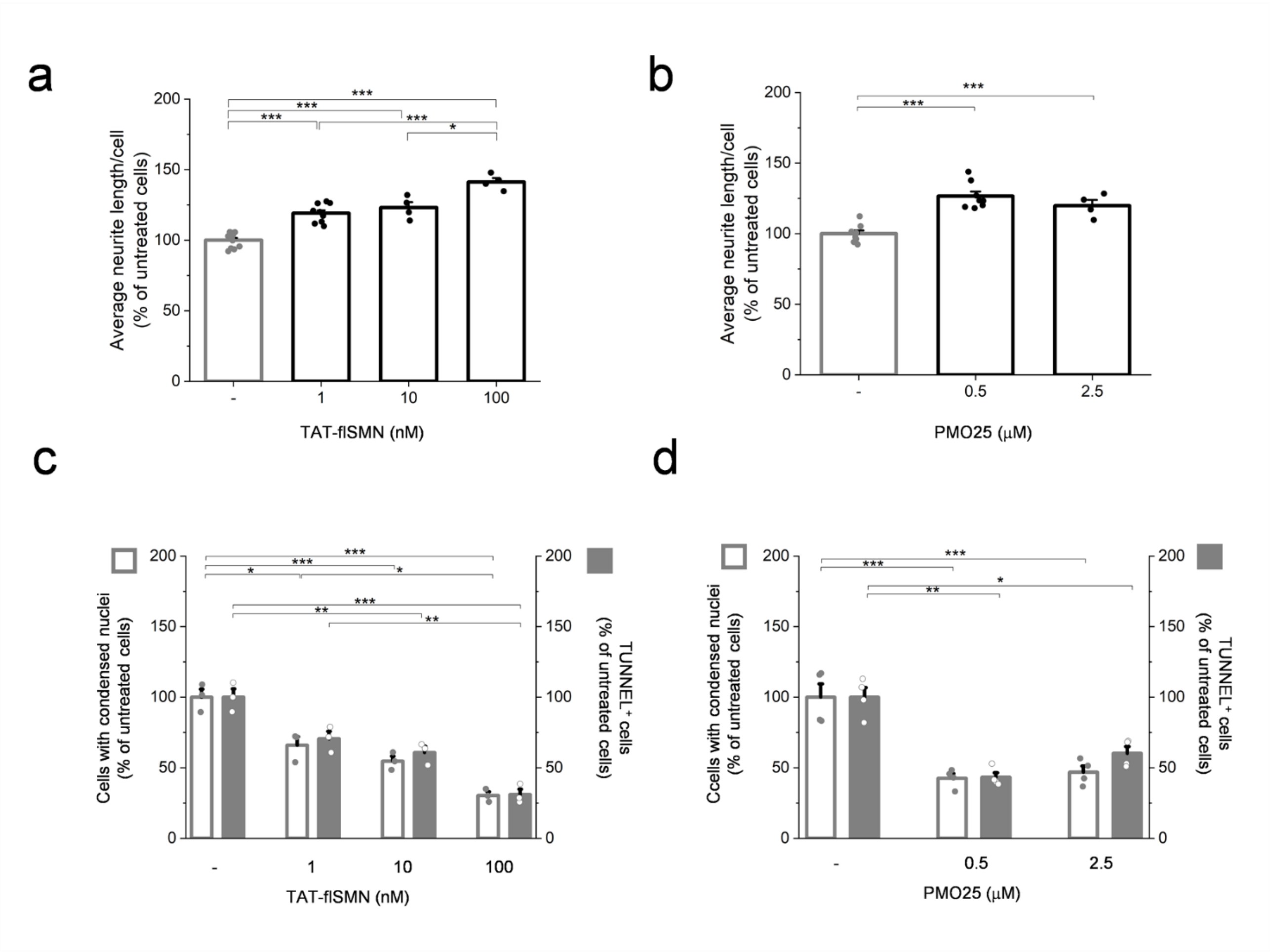


**Fig. S9. TAT-flSMN and PMO25 induce neurite elongation and prevent apoptotic cell death on SMA iPSC-derived MNs. a-b.** Quantitative analyses of the average neurite length per cell in SMA iPSC-derived MNs treated for 24 hours with increasing concentrations of TAT-flSMN **(a)** or PMO25 **(b)**. Data (mean ± SEM) are expressed as % in untreated conditions (85.31 ± 1.80 μm; $n$ = 4-12 in **(a)**; 87.27 ± 2.00 μm; $n$ = 4-8 in **(b)**. *$p<0.05$, **$p<0.01$, ***$p<0.0001$; Bonferroni-corrected one-way ANOVA. **c-d.** Quantitative assessments of SMA iPSC-derived MNs with condensed nuclei or TUNEL$^+$ upon treatment for 24 hours with increasing concentrations of TAT-flSMN **(c)** or PMO25 **(d)**. Data (mean ± SEM) are expressed as % in untreated conditions (% of cells with condensed nuclei: 4.67 ± 0.43%; % of TUNEL$^+$ cells: 4.25 ± 0.41%; $n$ = 3 in **(c)**; % of cells with condensed nuclei: 3.02 ± 0.35%; % of TUNEL$^+$ cells: 2.73 ± 0.32%; $n$ = 4 in **(d)**. *$p<0.05$, **$p<0.01$, ***$p<0.0001$, Bonferroni-corrected one-way ANOVA.

**Table S1. Summary of SAXS data analysis**.

| | |
|---|---|
| Data Collection | |
| Sample concentration (mg/ml) | 3.0 |
| SEC column | Spdx 75 3.2/300 |
| Beamline | ESRF BM29 |
| Beam energy (keV) | 12.5 |
| Sample-detector distance (m) | 2.867 |
| Exposure time (s) | 1.0 |
| Sample cell thickness (mm) | 1.0 |
| Temperature (°) | 20 |
| Final q range ($nm^{-1}$) | 0.01 – 3.00 |
| Data Analysis | |
| Points used for Guinier analysis | 12-27 |
| Guinier $qR_g$ limits | 0.74-1.27 |
| Guinier $R_g$ (nm) | 7.98 ± 0.09 |
| I(0) ($mm^{-1}$) | 113.59 ± 0.37 |
| $D_{max}$ (nm) | 29.69 |
| MW estimation ($V_c$ based) (kDa) | 480 |
| SASBDB ID | SASDRP5 |

# Supplementary Materials and Methods

## Therapeutic molecules

The recombinant TAT-flSMN fusion protein, harbouring N-terminal 6xHis and GST tags, was produced in *Escherichia coli* and subsequently purified to homogeneity by GenScript USA Inc. It was either purified as such or without tags and stored at -80° C according to the manufacturer's recommendations. Sample quality was verified through differential scanning fluorimetry (DSF) through a Tycho NT.6 instrument (NanoTemper Technologies GmbH, Munich, Germany). Fluorescein labelling was carried out by incubating TAT-flSMN at 0.05 mg/ml with NHS-fluorescein (Thermofisher, Waltham, MA) dissolved in dimethylsulfoxide (DMSO) in a 1:25 molar ratio in phosphate buffer saline (PBS) for 60 minutes at room temperature (RT), followed by desalting using a PD-minitrap G-25 equilibrated with PBS. Labeling efficiency was assessed by calculating the degree of labelling (DOL), defined as:

$$DOL = \frac{Abs_{493}}{\varepsilon_{fluor} \times \left(\frac{Abs_{280} - (Abs_{493}\ \times 0.3)}{\varepsilon_{protein}}\right)}$$

by measuring the sample absorbance using a NP80 (IMPLEN) microvolume spectrophotometer and using $e_{fluor}$ = 70'000 $M^{-1} \cdot cm^{-1}$ according to manufacturer's instructions, and $e_{protein}$ = 45'420 M-1·cm-1 as computed from Expasy ProtParam (1) using the TAT-flSMN sequence. For labelled TAT-flSMN used in this work, DOL values were consistently higher than 0.8.

The morpholino antisense oligonucleotide PMO25 (2), targeted against the intronic splicing silencer N1 (ISS-N1), was synthesized and purified by Gene Tools LLC. PMO25 was later dissolved to a standard concentration (1 mM) and stored according to the manufacturer's instructions.

## Sample preparation for LC-MS/MS analysis

Recombinant TAT-flSMN protein was precipitated by the addition of 10% (v/v) of an aqueous solution containing 2.7 M trichloroacetic acid (TCA). The resulting mixture was placed in ice for 2 hours followed by centrifugation at 14'000 rpm for 30 min. The supernatant was discarded and the protein pellet was dried by lyophilization and subsequently dissolved in 100 mM ammonium bicarbonate buffer pH 8.5 (95 µl); 5 µl of 100 mM dithiothreitol (DTT) solution were then added for performing the reduction of disulfide bonds (30 min at 60 °C). Finally, the sample was conditioned at 37 °C and a solution of trypsin (100 ng/µl, Promega, Madison,WI) was added to a final protein/enzyme ratio 50:1. Digestion was performed incubating overnight at 37 °C and the reaction was stopped by addition of Formic acid (FA) (2 µl). The resulting peptide mixture was separated and analysed as indicated in the following section.

## LC-MS/MS conditions

Analyses were carried out on an LC-MS (Thermo Finnigan, San Jose, CA) system consisting of a thermostated column oven; a Surveyor autosampler controlled at 25 °C; a quaternary gradient Surveyor MS pump equipped with an UV/Vis detector and an Ion Trap (LCQ Fleet) mass spectrometer with electrospray ionization ion source controlled by Xcalibur software 2.0.7. Analytes were separated by RP-HPLC on a Jupiter (Phenomenex, Torrance, CA) $C_{18}$

column (150 x 2 mm, 4 µm, 90 Å particle size) using a linear gradient (2–60% solvent B in 60 min) of solvent A (0.1% aqueous FA) and solvent B (0.1% FA in acetonitrile). Flow-rate was kept constant at 0.2 ml/min. Mass spectra were generated in positive ion mode under constant instrumental conditions: source voltage 5.0 kV, capillary voltage 46 V, sheath gas flow 20 (arbitrary units), auxiliary gas flow 10 (arbitrary units), sweep gas flow 1 (arbitrary units), capillary temperature 200 °C, tube lens voltage –105 V. MS/MS spectra, obtained by CID studies in the linear ion trap, were performed with an isolation width of 3 Th *m/z*, the activation amplitude was 35% of ejection RF amplitude, corresponding to 1.58 V. Data processing was performed using Peaks Studio version 4.5. The mass list was searched against the SwissProt protein database under continued mode (MS plus MS/MS) with the following parameters: trypsin specificity; five missed cleavages; peptide tolerance at 0.2 Da and MS/MS tolerance at 0.25 Da; peptide charge 1, 2, 3+ and experimental mass values: monoisotopic.

**Analytical size exclusion chromatography coupled to Small-Angle X-ray Scattering (SEC-SAXS)**

Recombinant TAT-flSMN was concentrated using Vivacon centrifugal filters (3 kDa cutoff, Sartorius, Göttingen, Germany), reaching a final concentration of 3 mg/ml. The sample was then injected into a Superdex 75 Increase PC 3.2/300 column (GE Healthcare, Chicago, IL), pre-equilibrated in 50 mM Tris/HCl, 500 mM NaCl, pH 8.0 and connected to an online Nexera HPLC system (Shimadzu, Kyoto, Japan) set with a flow rate of 0.075 ml/min at the BM29 small-angle X-ray scattering (SAXS) beamline of the European Synchrotron Radiation Facility (ESRF), Grenoble (3). Solution scattering data were collected using a frame rate of 1/sec. SEC-SAXS data were processed with *CHROMIXS* (4) for the evaluation of peak regions and buffer subtraction. Kratky analysis, extrapolation of radii of gyration and molecular masses were performed using PRIMUS (5). Results of SEC-SAXS analysis were deposited in the SADBDB database (entry id. SASDRP5) and are summarized in Table S1.

**Cell culture lines**

Mouse NSC-34 motor neuron-like cells (kindly provided by Prof. Neil R. Cashman, University of British Columbia, Vancouver, Canada) (6) were maintained in Dulbecco's modified Eagle's medium (DMEM, Gibco) supplemented with 10% fetal bovine serum (FBS, Sigma), 2 mM glutamine, 1 mM sodium pyruvate and 1% antibiotic/antimycotic solution (all others from Euroclone). NSC-34 cells were stably transfected with the psiRNA-hH1neo G2 control vector (InvivoGen) or the sh_Smn_519 silencing vector (kindly provided by Prof. Rashmi Kothary, The Ottawa Hospital Research Institute, Ottawa, Canada) (7) using FuGENE® HD Transfection Reagent (Promega) according to the manufacturer's instructions. Clonal colonies were isolated and cell lines were established for both constructs. The expression levels of SMN protein were then analyzed by Western blot. To induce NSC-34 differentiation into mature neurons, cells were plated onto poly-D-lysine coated coverslips in 24-well plates (5-10 x $10^3$ cells/well). After 48 hours, cells were subjected to differentiating conditions (DMed: 1:1 DMEM plus Ham's F12, 1% FBS, 1% antibiotic/antimycotic solution, and 1% modified Eagle's medium nonessential amino acids) (8).

**Human fibroblasts and induced pluripotent stem cell (iPSC)-derived motor neurons**

Human fibroblasts (GM00232, GM09677, GM03813) from SMA patients were obtained from the Coriell Institute for Medical Research and maintained in Minimal Essential Medium (MEM, Sigma, Burlington, MA) with 15% FBS (Sigma), 1% L-Glutamine, 1% Glucose, 1% antibiotic/antimycotic solution, 1% nonessential amino acid (all others from Euroclone, Milan, Italy).

Human induced pluripotent stem cells (CTRL iPSCs: GM24474*D; SMA iPSCs: GM24468*D) were obtained from the Coriell Institute for Medical Research, maintained on hESC-qualified Geltrex™-coated plates (Gibco, Waltham, MA) in TeSR™-E8™ medium (StemCell, Vancouver, Canada) and passaged every 3-4 days with Dispase II (1 mg/ml, Roche, Basel, Switzerland). Differentiation of human iPSC lines into mature motor neurons (MNs) was performed as described in Du *et al.* 2015 (9), with some modifications. Briefly, the day after the dissociation (DIV1) with Dispase II, TeSR™-E8™ medium was replaced with a defined neuronal medium composed as follows: DMEM/F12 and Neurobasal medium at 1:1, 0.5x N2 supplement, 0.5x B27 supplement, 0.1 mM ascorbic acid (Santa Cruz), 1x Glutamax, 1x antibiotic/antimycotic solutions (all others from Life Technologies, Carlsbad, CA). Medium was supplemented with 3 small molecules, i.e. 3 μM CHIR99021 (CHIR, Tocris, Bristol, United Kingdom), 2 μM DMH1 (DMH, Tocris), and 2 μM SB431542 (SB, Stemgent, Bestville, MD), and was changed every other day until DIV7. At DIV8, $SOX1^+$ NeuroEpithelial Progenitors (NEPs) were attained and dissociated with Dispase II, split at 1:6 on Geltrex™-coated plates and maintained in the same neuronal medium described above, but with the addition of 1 μM CHIR99021, 2 μM DMH1, 2 μM SB431542, 0.1 μM Retinoic Acid (RA, Stemgent) and 0.5 μM Purmorphamine (PUR, Stemgent). Medium was replaced on alternate days until DIV14. At DIV15, $OLIG2^+$ Motor Neuron Progenitors (MNPs) thus obtained were dissociated with Dispase II, split at 1:20-1:40 on Geltrex™-coated plates and expanded in the above neural medium, containing 0.5 μM RA and 0.1 μM PUR. Medium was changed every second day for 7 days (DIV15-21) to obtain beta III-tubulin (TUJ1)-, Islet-1- and MNX1-immunopositive MNs. At DIV22, $MNX1^+$ MNs were dissociated with Accumax (StemCell) into single cells and suspended in neural medium additioned with 0.5 μM RA, 0.1 μM PUR, and 0.1 μM Compound E (StemCell), a NOTCH inhibitor to block progenitor proliferation. A mixture of growth factors (GFs), i.e. BDNF, IGF-1, and CTNF (10 ng/ml, all from Peprotech, Cranbury, NJ), was added to the medium after 24 hours, to promote the maturation process. Medium was replaced every three days until choline acetyltransferase (ChAT)-immunopositive MNs were attained.

**Quantitative RT-PCR**

Total RNA was extracted from human fibroblasts using ReliaPrep™ RNA Cell Miniprep System (Promega) according to the manufacturer's guidelines. 1 µg of total RNA was reverse-transcribed using iScript cDNA Synthesis Kit (Bio-Rad) according to manufacturer's instructions. A total of 10 ng of cDNA was used as a template for quantitative PCR performed with the SsoFast EvaGreen Supermix (Bio-Rad, Hercules, CA) on a CFX96 Real-Time PCR Detection System (Bio-Rad). Relative expression of human *SMN2* and human *SMNΔ7* was determined by the $2^{-\Delta\Delta ct}$ method and normalized to the housekeeping genes hypoxanthine guanine phosphoribosyl transferase (*HPRT*). Primer sequences are provided upon request.

**Western blotting analysis**

Forty-eight hours after seeding, total protein was extracted from A.1 CTRL, GM.5, and C.2 clonal cell lines using a lysis buffer composed of 0.5% NP40, 0.5% sodium deoxycholate in PBS pH 7.4, supplemented with Protease Inhibitor Cocktail Tablets Complete Mini (Roche). Protein concentration was quantified using a Pierce® BCA Protein Assay Kit (Thermo Fisher Scientific). 10 µg of total protein were resolved in 12.5% SDS-polyacrylamide gel and transferred onto a nitrocellulose membrane (Whatman, Little Chalfont, United Kingdom). The membrane was stained with Ponceau S (Sigma) to visualize the protein bands and then rinsed with distilled water. After blocking with 5% milk in TBST buffer, the membrane was incubated with a monoclonal anti-SMN antibody (mouse, 1:10000, BD

Bioscences, Franklin Lakes, NJ) or a monoclonal anti-β-actin antibody (mouse clone AC-15, 1:5000, Sigma) for 1 hour at room temperature (RT). After washing in TBST buffer, the membrane was incubated with an anti-mouse IgG peroxidase secondary antibody (1:3000, Sigma) for 1 hour. Membranes were rinsed and incubated with SuperSignal West Pico PLUS chemiluminescent Substrate (Thermo Fisher) for 5 minutes. Full-blot scans were taken with ChemiDoc™-XRS (Bio-Rad). Densitometrical analysis of the bands was performed with Image Lab™ software system ver. 5.1 (Bio-Rad) and SMN/β-actin ratios were calculated.

**Electrophysiology**

Whole-cell patch-clamp experiments were performed at room temperature on CTRL iPSC-derived MNs at ~ DIV30. Cells were visualized by using an Eclipse TE200 (Nikon) inverted microscope, as previously described (10). During electrophysiological recordings the extracellular solution contained (in mM): 140 NaCl, 1 $MgCl_2$, 2 $CaCl_2$, 3 KCl, 10 glucose, 10 HEPES (pH 7.4 with NaOH). Recording pipettes were produced from borosilicate glass capillary tubes by means of a horizontal puller (P-97, Sutter instruments, Novato, CA) and filled with an intracellular solution containing (in mM): 130 potassium gluconate, 4 NaCl, 2 $MgCl_2$, 1 EGTA, 10 HEPES, 5 creatine phosphate, 2 $Na_2$-ATP, 0.3 $Na_3$-GTP (pH 7.3 with KOH). Membrane voltage was corrected off-line for a calculated liquid junction potential of -10 mV. Series resistance was monitored throughout the experiment. Recordings were made with an Axopatch 200B amplifier (Axon Instruments, San Jose, CA) and digitized with a Digidata 1322A AD/DA converter (Axon Instruments). Signals were acquired using Clampex 10.2 (Molecular Devices, San Jose, CA), sampled at 20 kHz, and filtered at 10 kHz. Software Clampfit 10.2 (Molecular Devices) and Origin (Microcal, Malvern, United Kingdom) were used for data analysis.

Cell capacitance (Cm) was calculated by integrating the capacitive current evoked by a −10 mV pulse. Resting membrane potential (Vm) was measured as soon as the whole-cell configuration was reached whereas neuronal input resistance (Rin) was calculated in the linear portion of the I–V relationship during hyperpolarizing voltage responses near the resting potential. Activation of inward and outward currents mediated respectively by voltage-dependent sodium and potassium channels was achieved by stimulating the cell with steps of voltage of increasing amplitude. Firing properties were assessed by recording the voltage responses to a series of supra-threshold current steps starting from a holding potential of -70 mV. The threshold of the action potentials ($AP_{Th}$) was computed as the value of the membrane potential at which a rapid upstroke of the action potential starts. The amplitude of the action potentials ($AP_A$) was measured as the voltage difference between the peak of the spike and the $AP_{Th}$. The AP duration ($AP_D$) was calculated as the spike width measured at half-maximal $AP_A$. Action potentials were considered abortive when $AP_A$ was lower than 40 mV. The expression of GABA-A and AMPA receptors on the cell membrane was demonstrated by measuring the current evoked by direct application of 10 µM GABA and/or 200 µM AMPA on the recorded at a holding potential of 0 mV and -70 mV, respectively.

**Transfection and Luciferase Reporter Assay**

NSC-34 cells (2 x $10^4$ cells/well) were grown in 24-well plates and transiently transfected with the pCEP4-*SMN1*-luc or pCEP4-*SMN2*-luc plasmids (kindly gifted by Prof. Androphy E.J., Indiana University School of Medicine, Indianapolis, United States) (11). Transfection was performed with FuGENE® HD Transfection Reagent (Promega) according to the manufacturer's instructions. Twenty-four hours after the transfection, cells were incubated in the absence or in the presence of increasing concentrations of PMO25 (0.1, 0.5, 1.5, 2.5, 7.5, 22.5 µM; Gene Tools LLC)

for 48 hours. Cells were subsequently lysed by using a Passive Lysis Buffer (Promega) and luciferase enzymatic activity was determined. Firefly and *Renilla* luciferase signals were measured using a Dual-Luciferase Reporter Assay System (Promega) according to manufacturer's instructions, through a GloMax® Explorer System (Promega). The normalized firefly luminescence/*Renilla* luminescence ratio was calculated per each sample.

**Immunocytochemistry**

Immunostainings were performed as follows. Cells were washed once in PBS before fixation with 4% PFA for 20 minutes at RT. After fixation, cells were stained with Hoechst 33342 (5 µg/ml, Sigma) for 15 minutes and, then, permeabilized with 0.2% Triton X-100 (Sigma) in PBS at RT for 5 minutes. Blocking was performed with 5% of the relevant Normal Serum (Sigma) and 0.1% Triton X-100 in PBS for 1 hour at RT. Primary antibodies were diluted in blocking solution and incubated overnight at 4°C. The following day, cells were carefully washed and incubated with secondary antibodies diluted in blocking solution for 2 hours at RT.

***In vitro* treatments**

To assess the ability of TAT-flSMN to penetrate into cells, NSC-34 motor neuronal cells were plated either in 6-well plates ($1 \times 10^5$ cells/well) or in 24-well plates containing glass coverslips ($2 \times 10^4$ cells/well). For LC-MS/MS analysis, cells were incubated with 100 nM TAT-flSMN for 48 hours, washed with ice-cold phenol-free PBS, transferred in a tube, and centrifuged at 1'000 g for 10 min at 4°C. The resulting supernatant was discarded and cell pellet was stored until use. For fluorescein-labelled protein detection, cultures were treated with 10 µg/ml Hoechst 33342 (Sigma) for 15 min, washed, and incubated for 48 hours with 100 nM TAT-flSMN either unlabelled or labelled with fluorescein. The presence of the proteins inside the cells was evaluated by a digital camera (DFC 310 FX, Leica Microsystem, Wetzlar, Germany) mounted on a DM5000 B microscope (Leica Microsystem) and analyzed by Leica Application Suite 3.8.0 software.

To study the axonotrophic and neuroprotective activities of the TAT-flSMN protein on the SMN knock-down NSC-34 clonal cell lines, GM.5, and C.2 cells were plated onto poly-D-lysine-coated coverslips in 24-well plates (5-10 x $10^3$ cells/well). Forty-eight hours after seeding, cells were pre-incubated for 3 hours with increasing protein concentrations (1, 10, 100 nM; GenScript USA Inc.). Cells were then subjected to DMed and maintained in the presence of TAT-flSMN or saline. After 48 hours, they were fixed with 4% PFA.

To investigate the axonotrophic and neuroprotective activity on iPSC-derived MNs of TAT-flSMN and PMO25, individually or in combination, both CTRL iPSCs and SMA iPSCs were plated (1 x $10^5$ cells/well) on Geltrex™-coated coverslips in 24-well plates and subjected to the previously described differentiation protocol. At DIV29 of MN differentiation, CTRL iPSC- and SMA iPSC-derived MNs were treated with increasing concentrations of TAT-flSMN (1, 10, 100 nM; GenScript USA Inc.) or PMO25 (0.5- 2.5- 7.5 µM; Gene Tools LLC). After 24 hours, each treatment was stopped by fixing the cells with 4% PFA.

**Sample preparation and nano-ESI-LC-MS/MS conditions used for protein internalization analysis**

NSC-34 cell samples were lysed, proteolyzed following the filter-aided sample preparation (FASP) protocol (12) and analyzed via LC-MS/MS on an UltiMate 3000 RSLC nano-HPLC system (Thermo Fisher Scientific) that was coupled to a timsTOF Pro mass spectrometer equipped with CaptiveSpray source (Bruker Daltonics). Peptides were trapped on a C18 column (precolumn Acclaim PepMap 100, 300 µm × 5 mm, 5 µm, 100 Å, Thermo Fisher Scientific)

at 50°C and separated on a self-packed Picofrit (New Objective, Littletone, MA) nanospray emitter (360 µm o.d. × 75 µm i.d. x 400 mm L, 15 µm Tip i.d.) with C18-stationary phase (3.0 µm, 120 Å, Dr. Maisch GmbH). After trapping, peptides were eluted (column temperature 40°C) by a linear 180 min water–acetonitrile (ACN) gradient from 3% (v/v) to 50% (v/v) ACN. The values for mobility-dependent collision energy ramping were set to 95 eV at an inverse reduced mobility (1/k0) of 1.6 V s/cm2 and 23 eV at 0.73 V s/cm2. Collision energies were linearly interpolated between these two 1/k0 values and kept constant above or below. No merging of TIMS scans was performed. Target intensity per individual PASEF precursor was set to 20000. The scan range was defined between 0.6 and 1.6 V s/cm2 with a ramp time/accumulation time of 166 ms. 14 PASEF MS/MS scans were triggered per cycle (2.57 s) with a maximum of seven precursors per mobilogram. Precursor ions in an m/z range between 100 and 1700 with charge states ≥ 2+ and ≤ 8+ were selected for fragmentation. Active exclusion was enabled for 0.4 min (mass width 0.015 Th, 1/k0 width 0.015 V s/cm2).

**Data analysis performed to evaluate protein internalization**

Protein identification was performed with MaxQuant v2.5 (13). The following settings were applied: proteolytic cleavage C-terminally at Lys and Arg, up to 3 missed cleavages were allowed; minimum peptide length: 6 amino acids; modifications: alkylation of Cys by iodoacetamide (fixed), oxidation of Met (variable); acetylation of protein N-terminus (variable); precursor mass accuracy: 10 ppm; fragment ion mass accuracy: 20 ppm; false discovery rate (FDR) cut-off: 1%. The sequence of the exogenous protein was added to the FASTA file containing the Mus Musculus proteome (Swiss-Prot UP000000589).

**Morphometric analysis**

Fixed SMN knock-down NSC-34 cells were labelled with Hoechst 33342 (5 µg/ml, Sigma) and anti-TUJ1 (mouse, 1:500, Sigma) antibody. Fixed iPSC-derived MNs were stained with Hoechst 33342 and immunolabelled with anti-ChAT (goat, 1:50, Chemicon) and anti-TUJ1 (mouse, 1:500, Sigma) antibodies. After rinsing, coverslips with seeded cells were mounted using fluorescence mounting medium (Dako, Glostrup, Denmark). For each experiment, two different coverslips were stained and images acquired (5 fields/coverslip, 10 fields in total) with a 20X objective. Analysis of neurite length was performed using the NeuronJ plugin from the ImageJ package Fiji (1.52n, Wayne Rasband, NIH http://imagej.nih.gov/ij). Neurites from valid cell bodies, i.e. immunopositive for TUJ1 for NSC-34 cells and immunopositive for TUJ1/ChAT for differentiated iPSC-derived MNs, were quantified as follows. For NSC-34 clonal cell lines, the number of cells without neurites and the mean length of the longest neurite per TUJ1-immunopositive cell were considered. For differentiated iPSC-derived MNs, after tracing all neurites, the average neurite length per TUJ1/ChAT-immunopositive cell was calculated.

**Cell death assay**

Fixed SMN knock-down NSC-34 cells were incubated with Hoechst 33342 (5 µg/ml, Sigma) to detect condensed nuclei, the fluorogenic caspase substrate DEVD-NucView488 (5 µM, Biotium, Fremont, CA) to detect caspase-3 activation, and an antibody for the pan-neuronal marker TUJ1 (mouse 1:500, Sigma) to identify neurons. Fixed iPSC-derived MNs were labelled with Hoechst 33342, DeadEnd™ Fluorometric TUNEL System (Promega, according to the manufacturer's guidelines) and TUJ1 to detect nuclear condensation, nuclear DNA fragmentation, and to identify neurons, respectively. After rinsing, coverslips with seeded cells were mounted using fluorescence mounting medium

(Dako). The number of cells positive for the active caspase 3 and/or showing condensed and/or TUNEL$^+$ nuclei was counted in 10 microscopic fields (40X) per coverslip. In each experiment, 2 coverslips/condition were counted. To determine motor neuronal cell death, the percentage of cells positive for active caspase 3 and/or exhibiting condensed and/or TUNEL$^+$ nuclei was calculated on the total number of TUJ1-positive cells.

**Statistical analysis**

Data are represented as mean ± SEM or SD and statistical significance was verified using OriginPro 2018 software. Two-tailed unpaired *t*-test was used for comparisons between two groups; Bonferroni-corrected one-way ANOVA was used for comparisons of multiple groups; Bonferroni-corrected repeated measures two-way ANOVA was used for behavioral tests; the Logrank test was used for survival analysis.

**References**


1. Gasteiger E, Hoogland C, Gattiker A, Duvaud S, Wilkins MR, Appel RD, et al. Protein Identification and Analysis Tools on the ExPASy Server. In: Walker JM, editor. The Proteomics Protocols Handbook. Springer Protocols Handbooks: Humana Press; 2005. p. 571 - 607.
2. Zhou H, Janghra N, Mitrpant C, Dickinson RL, Anthony K, Price L, et al. A novel morpholino oligomer targeting ISS-N1 improves rescue of severe spinal muscular atrophy transgenic mice. Hum Gene Ther. 2013;24(3):331-42.
3. Pernot P, Round A, Barrett R, De Maria Antolinos A, Gobbo A, Gordon E, et al. Upgraded ESRF BM29 beamline for SAXS on macromolecules in solution. J Synchrotron Radiat. 2013;20(Pt 4):660-4.
4. Panjkovich A, Svergun DI. CHROMIXS: automatic and interactive analysis of chromatography-coupled small-angle X-ray scattering data. Bioinformatics. 2018;34(11):1944-6.
5. Konarev PV, Volkov VV, Sokolova AV, Koch MHJ. Svergun DI. P*RIMUS* : a Windows PC-based system for small-angle scattering data analysis. Journal of Applied Cystallography. 2003;36:1277-82.
6. Cashman NR, Durham HD, Blusztajn JK, Oda K, Tabira T, Shaw IT, et al. Neuroblastoma x spinal cord (NSC) hybrid cell lines resemble developing motor neurons. Dev Dyn. 1992;194(3):209-21.
7. Shafey D, Côté PD, Kothary R. Hypomorphic Smn knockdown C2C12 myoblasts reveal intrinsic defects in myoblast fusion and myotube morphology. Exp Cell Res. 2005;311(1):49-61.
8. Eggett CJ, Crosier S, Manning P, Cookson MR, Menzies FM, McNeil CJ, et al. Development and characterisation of a glutamate-sensitive motor neurone cell line. J Neurochem. 2000;74(5):1895-902.
9. Du ZW, Chen H, Liu H, Lu J, Qian K, Huang CL, et al. Generation and expansion of highly pure motor neuron progenitors from human pluripotent stem cells. Nat Commun. 2015;6:6626.
10. Conforti P, Bocchi VD, Campus I, Scaramuzza L, Galimberti M, Lischetti T, et al. -derived medium spiny neurons recapitulate human striatal development and complexity at single-cell resolution. Cell Rep Methods. 2022;2(12):100367.
11. Cherry JJ, Evans MC, Ni J, Cuny GD, Glicksman MA, Androphy EJ. Identification of novel compounds that increase SMN protein levels using an improved SMN2 reporter cell assay. J Biomol Screen. 2012;17(4):481-95.
12. Wiśniewski JR, Zougman A, Nagaraj N, Mann M. Universal sample preparation method for proteome analysis. Nat Methods. 2009;6(5):359-62.

13. Tyanova S, Temu T, Cox J. The MaxQuant computational platform for mass spectrometry-based shotgun proteomics. Nat Protoc. 2016;11(12):2301-19.
